# The efficacy of transcranial direct current stimulation in the treatment of anorexia nervosa: a randomized double-blind clinical trial

**DOI:** 10.3389/fpsyt.2024.1284675

**Published:** 2024-05-02

**Authors:** Zuzanna Rząd, Joanna Rog, Natalia Kajka, Paweł Szewczyk, Paweł Krukow, Hanna Karakuła-Juchnowicz

**Affiliations:** ^1^ Department of Psychiatry, Psychotherapy and Early Intervention in Lublin, Medical University of Lublin, Lublin, Poland; ^2^ Laboratory of Human Metabolism Research, Department of Dietetics, Institute of Human Nutrition Science, Warsaw University of Life Sciences (WULS-SGGW), Warsaw, Poland; ^3^ Department of Clinical Neuropsychiatry, Medical University of Lublin, Lublin, Poland

**Keywords:** anorexia nervosa, transcranial direct current stimulation, eating disorders, brain stimulation, neurostimulation, tDCS

## Abstract

**Background:**

Anorexia nervosa (AN) is a life-threatening disease with a low effectiveness of treatment. The high relapse and mortality rate indicate new treatment approaches are needed. Here, we represent the protocol for randomized clinical trial (RCT) of transcranial direct current stimulation (tDCS) efficiency in the AN treatment. The main purpose of the 3-week RCT is to determine the effect of tDCS on the mental state and advances in nutritional rehabilitation in patients with AN.

**Methods:**

50 female inpatients (13-25 years old, body mass index (BMI) 17.5 kg/m2 or less) will be randomly allocated into groups: active (n=25) and sham (n=25) tDCS. Thirty 25-minute tDCS sessions (applied current at 2mA) will be given to DLPFC (F3 anode/F4 cathode) twice a day for 3 weeks on working days parallel to treatment as usual. The primary outcome measures include changes in symptoms related to eating disorders, as assessed by the Eating Attitudes Test (EAT-26), following tDCS sessions over a 3-week trial period. The secondary outcome measures include changes in: brain bioelectric activity, anthropometric measurements, mood, nutritional status, neurocognition, psychological symptoms, selected biological markers related to stress, food intake, inflammation and neurotrophins.

**Discussion:**

This paper describes the evaluation of a 3-week tDCS-based intervention for AN patients. The study design was developed by a multidisciplinary research team to assess the treatment effect, taking into account various types of variables. This approach could help in better understanding the potential therapeutic tDCS strategy in AN.

**Clinical trial registration:**

www.ClinicalTrials.gov, identifier NCT05814458.

## Introduction

### Background and rationale

Anorexia nervosa (AN), a form of eating disorder, is identifiable through several characteristic symptoms. These encompass the adoption of a restrictive diet leading to weight loss, a distorted perception of one’s body, the fear of gaining weight, decreased libido, multidimensional cognitive dysfunction, and the implementation of hedging and avoidance strategies (1). AN, recognized as a life-threatening condition, transcends global boundaries, affecting individuals across diverse socio-economic strata. Its prevalence is reported to be between 0.3-0.6% in women and 0.1% in men (2). The intricate nature of AN treatment is marked by numerous complications, resulting in a significant number of deaths. Particularly alarming is the mortality rate among girls and women aged 15 to 24, reported to be 12 times higher than the overall mortality rate from all causes combined (3). Various treatment modalities are employed, encompassing nutritional rehabilitation, individual psychotherapy, family-based therapy, nutritional counseling, and pharmacotherapy (SSRIs) (4–6). Currently, there are no licensed or approved biological treatments for AN, and the effectiveness of psychological interventions and weight restoration treatments varies and typically leads to temporary alleviation of symptoms in around one-third of adolescent patients, with an even lower rate of success in adults (7). Risk factors for AN can be presented in three groups: temperamental/personality, environmental, genetic and physiopathological (8).

The existing literature highlights the presence of brain abnormalities in individuals with AN, particularly in specific areas such as the dorsolateral prefrontal cortex (DLPFC), cingulate cortex, and left middle occipital gyrus (9). Electroencephalographic (EEG) measurements among patients suffering from AN have shown hyperactivity of the right frontal hemisphere (10). Functional magnetic resonance imaging studies reveal diverse patterns of brain activity. Notably, when exposed to food cues, there is reported heightened activity in regions associated with reward, along with increased activation in the right DLPFC (11). These findings suggest an intensified top-down inhibition of reward processing (12). Importantly, the DLPFC is recognized for its pivotal role in cognitive control and self-regulation in dietary contexts (13), therefore, it is reasonable to question whether coupled inhibition of the right hemisphere activity can facilitate interhemispheric balance (14).

Due to the complex etiology of AN and the limited effectiveness of current therapies, coupled with a high mortality rate, there has been a search for new treatment methods. Contemporary research directions in the therapy of AN increasingly focus on neuromodulation and neurosurgery, encompassing neurosurgical ablation, deep brain stimulation, repetitive transcranial magnetic stimulation, and transcranial direct current stimulation (tDCS) (15). In our project, we formulated a hypothesis regarding the effectiveness of tDCS in the treatment of anorexia, measured as a reduction of the severity of eating disorder symptoms. At the time of writing this article, there are also four published research papers (14, 16–18) and two study protocols (19, 20) assessing the effectiveness of such therapy in AN, which encompass diverse approaches and outcomes. As of now, only one double-blind randomized controlled trial (17) has delved into the therapeutic possibilities of tDCS for AN. In this particular study, 43 participants diagnosed with AN were randomly assigned to undergo either active or sham tDCS sessions targeting the left DLPFC over ten 30-minute sessions. While the findings did not reveal significant improvements in AN psychopathology or weight recovery compared to sham tDCS, active tDCS did demonstrate a decrease in the need for excessive control over food intake and an enhancement in body image.

TDCS method serves as a tool for brain stimulation, enabling the stimulation of the cerebral cortex through the use of two or more sponge electrodes with opposing polarities (anode and cathode). These electrodes, soaked in saline, are applied to the scalp (21). This procedure is painless, and considered safe, with minimal to no side effects (22). The stimulator, a battery-powered device, administers a small amount of direct current (typically 0.5–2.5mA), some of which reaches the brain, influencing brain activity based on the applied current’s polarity. tDCS provides two types of stimulation: anodal and cathodal (23). During a tDCS session, a current flows between the electrodes, resulting in non-focal effects. Anodal tDCS depolarizes neurons, increasing their excitability and facilitating firing, whereas cathodal tDCS hyperpolarizes neurons, inhibiting firing below the stimulation site. Typically, the target neurons become less excitable, leading to a decrease in their spontaneous activity (24). According to physiological studies conducted during stimulation, the mechanism of action of tDCS is multidirectional. Studies using positron emission tomography have identified potential mechanisms of action in the structures of the central nervous system of transcranial stimulation: the possibility that tDCS may act through the opioidergic system (increasing the release of endogenous opioids), acting through voltage-dependent sodium channels, dopamine type two (D2) receptors, and through serotonergic (5-HT) receptors. It has also been observed that transcranial stimulation is followed by NMDA receptor activation and a subsequent process of long-term synaptic potentiation (25, 26). An increase in glutamatergic and GABA-ergic activity has also been observed (27).

The study assumed that tDCS treatment would directly or indirectly lead to changes in brain bioelectrical function in the left and right frontal lobe areas. Studies describing the efficacy of tDCS indicate improvements in neurocognitive function, mood, increases in serotonin and dopamine (28, 29). This will create an opportunity to implement more effective and new methods of recovery in the course of AN.

### Objectives

We hypothesized that applying excitatory tDCS could potentially help modify the balance between the two brain hemispheres in children and adolescents with AN. The aim is to diminish their control over eating behaviors and enhance overall improvements in AN psychopathology. Our primary outcome measure involves assessing changes in the Eating Attitudes Test (EAT-26) from baseline to end of stimulation cycle (3 weeks).

A range of secondary outcome measures will be administered to further determine tDCS treatment effects on: 1. anthropometric measurements (in this BMI), 2. brain bioelectric activity, 3. nutritional status, 4. mood, 5. neurocognition (attention, memory, and some aspects of planning, verbal fluency, and another executive function), 6. psychological measurements: dysfunctional metacognitive beliefs, other aspects related to thinking such as reflectivity, impulsivity, obsessive-compulsive symptoms, stress based on subjective feelings related to problems and personal events, 7. selected biological markers related to stress, food intake, inflammation and neurotrophins.

### Trial design

**Figure 1:**
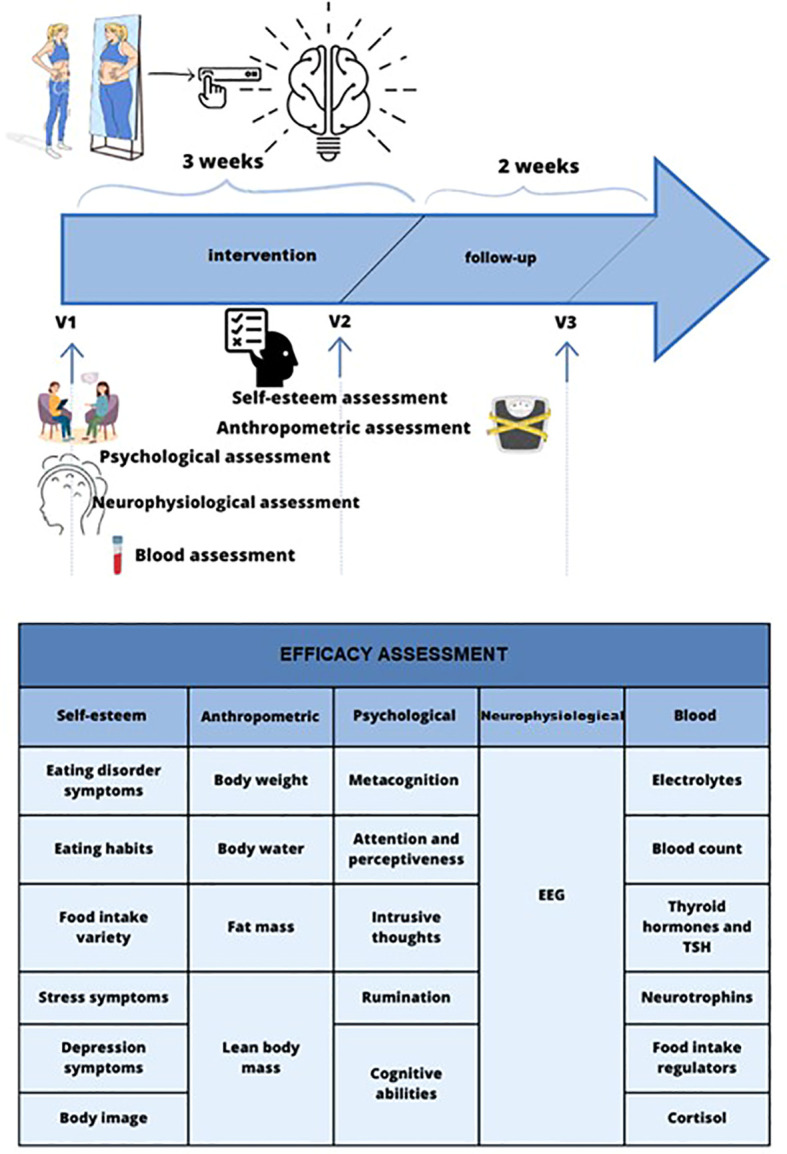
Visual representation of the study protocol.

In a single-center randomized, double-blind, placebo-controlled study (RCT), the effect of tDCS (n=25) compared to sham-tDCS (n=25) will be evaluated in patients suffering from AN. The aim is to assess the effectiveness of tDCS in enhancing the clinical outcomes for children and adolescents with AN. We will measure this improvement by evaluating changes in the scores of the EAT-26 (Eating Attitudes Test-26) as our primary outcome. The study was designed according to the guidelines Recommendations for Interventional Trials recommendations (SPIRIT 2013) (30). The duration of intervention was set for three weeks as a main endpoint and with follow-up period 2 weeks after the last tDCS session. The graphical representation of the overall study structure shows **Figure 1**. The RCT was registered in the clinicaltrials.gov database (study ID: NCT05814458).

## Methods: participants, interventions, and outcomes

### Study setting

Recruitment and study activities will be conducted among inpatients at the I Department of Psychiatry, Psychotherapy and Early Intervention at the Medical University of Lublin, Poland.

### Eligibility criteria

A total of 50 inpatients (before accounting for a 20% dropout) with a diagnosis of anorexia nervosa (AN) will be recruited for this study. To be eligible for the trial, subjects must fulfill all of the inclusion criteria and none of the exclusion criteria, as stated below. The following inclusion criteria will be adopted:

1. Written informed consent to participate in this study before any study-mandated procedure, written by the participant or participant and parent/legal guardian;
2. Female patients aged 13–25 years old;
3. Meet the DSM-5 criteria for AN;
4. Body mass index (BMI)  ≤ 17,5 kg/m2;
5. Participants must not be undergoing psychopharmacotherapy, with the exception of those who have been taking antidepressants for a minimum of 1 month in a stable dose.

The exclusion criteria will be as follows:

1. No informed consent to participate in the study;
2. Diagnosis of neurological/organic diseases, such as epilepsy;
3. Contraindications to tDCS, ie., pacemakers, metal parts around the head;
4. Any psychiatric comorbidities;
5. Pregnancy or pregnancy planning;
6. Taking medications other than antidepressants or changes in psychopharmacotherapy during the study.

Reasons for the participant to be discontinued from the study:

1. Withdrawal of informed consent,.
2. Completion of hospitalization before the end of participation in the project.
3. Non-attendance at the study visits and stimulations understood as equal to or greater than 10% of all simulations.
4. Any serious adverse event during the intervention period.

### Interventions

Patients will be receiving twice a day tDCS (applied current at 2mA) or sham stimulation, for 3 weeks on working days (30 sessions in total). Each stimulation will last 25 minutes and take place at a two-hour interval. The detailed schedule of meetings and measurement methods is presented in **Table 1**.

**Table 1:** Schedule of enrolment, interventions, and assessments.

	Study Period				
	Enrolment	Baseline	Intervention period (tDCS stimulations)	End of the intervention period	Safety Follow-up
Timepoint (day)	-2 to -1	0	1-21 ( ± 1 days)	22 ( ± 1 days)	35 ( ± 7 days)
Visit number	V0	V1		V2	V3
Eligibility screen	X				
Informed consent	X				
Sociodemographic data	X				
Randomization		X			
ASSESSMENTS:					
Weight	X	X	X	X	X
Performing body composition analysis		X		X	X
Psychological		X		X	
Neuropsychological		X		X	
Dietary behaviors and nutrition		X		X	X
Biological	X	X	X	X	
Adverse events	X	X	X	X	X

Participants in the active tDCS group will receive active stimulation to DLPFC via two saline-soaked, 25 cm2 (5 cm x 5 cm) sponges saline-soaked placed over F3 (anode) and F4 (cathode), according to the International 10–20 system. The electrodes will be placed on the patient’s head using a specially designed headband. The patient’s skin should be clean and dry. Each time, it will be checked whether the skin has any wounds, irritations, or other dermatological issues that could affect the safety and comfort of the patient.

In the sham tDCS condition, an identical protocol was employed. When the system is programmed as Sham, the device will ramp up to the programmed intensity and then back down to a test current of 25 microamps. It will stay at this test current to make sure the contact quality measurement is correct for the remainder of the session and then will ramp up to the programmed intensity and then back down to the test current at the end of the session. The system will ramp up for 30 seconds and back down for 30 seconds at the beginning and the end of each session for Sham stimulation. For Active stimulation, there is an initial ramp up of 30 seconds to the programmed intensity and then at the end of the session, the current ramp down to test current in 30 seconds. The commercially available Soterix Medical Inc. 1x1 tDCS mini-CT LTE device (model 1601-LTE) will be used for stimulation. The first stimulation will take place after obtaining the patient’s written consent, completing questionnaires, performing body composition analysis, EEG and blood tests. The stimulation device will always be triggered by one of the team members. During the stimulation, the patient can move freely and will be encouraged to do activities that additionally stimulate the brain, such as reading, solving crosswords or engaging in conversation. During the school year, simulations will take place during lessons conducted at the hospital. All patients will additionally receive treatment-as-usual, including nutritional rehabilitation, individual and family psychotherapy, psycheducation, and (if necessary) pharmacotherapy.

### Measures

To verify the effectiveness of both the active and sham tDCS stimulation methods, the following measures will be used:

I. For sociodemographic data: self-prepared personal questionnaire. The survey will cover personal data, medical history, information on menstruation, and used pharmacotherapy.

II. For psychological data:

The psychological examination consists of 14 tests using the paper-and-pencil method and is divided into 2 parts (the minimum interval between their administration is 4 hours, presented in the **Table 2**). During this time between examinations, patients were hospitalized in the ward. The psychological diagnosis will examine the following areas: neurocognitive functions (complex attention, executive functions, memory, language, social cognition), mood, obsessive-compulsive symptoms, and stress levels and assessment of early childhood injuries related to the experience of abuse, emotional, bodily, sexual, emotional, and physical neglect, and level of satisfaction with one’s own body. The first part includes the following tools: The Rey-Osterrieth complex figure test (ROCF), The Children’s Depression Inventory 2 (CDI-2)/the Beck Depression Inventory (BDI), The Yale-Brown Obsessive Compulsive Scale (CY-BOCS), The Color Connection Test (CTT1 and CTT2), The Benton Visual Retention Test (BVRT), The Perceived Stress Scale (PSS-10), The Childhood Trauma Questionnaire (CTQ) (30- 45 minutes); the second part includes: The Ruminance-Reflectivity Questionnaire (RRQ), The Attention and Perceptiveness Tests (APT), The Appearance Self-Rating Sheet (ASRS), The Verbal Fluency Test (VFT), The Metacognition Questionnaire (MCQ-A) (30- 45 minutes) measure for V1 and V2 respectively.

**Table 2:** List of psychological tests performed on patients with AN.

Part 1	Part 2
1. The Rey-Osterrieth complex figure test 2. The Children’s Depression Inventory 2/Beck Depression Inventory 3. The Yale-Brown Obsessive Compulsive Scale 4. The Color Connection Test 5. The Benton Visual Retention Test 6. The Perceived Stress Scale 7. The Childhood Trauma Questionnaire	1. The Matching Familiar Figures Test 2. The Attention and Perceptiveness Tests 3. The Verbal Fluency Test 4. The Appearance Self-Rating Sheet 5. The Metacognition Questionnaire 6. The Ruminance-Reflectivity Questionnaire

1) The Beck Depression Inventory (BDI) by Aaron Beck- It is a self-assessment tool that is helpful in screening the diagnosis of depression and measuring its severity (31). It consists of 21 multiple-choice questions. This tool will be used for adolescents who are over 18 years of age. The Polish adaptation was validated by Zawdzki, Popiel & Pragłowska. Cronbach’s alpha was 0.930 and 0.950, respectively (32);
2) The Children’s Depression Inventory 2 (CDI2) by Maria Kovacs. A set of questionnaires is responsible for the self-reporting measurement of the severity of symptoms of depression in children and adolescents. It consists of 28 questions, the respondent chooses the answer containing the mode of functioning closest to his or her well-being. The filling time is 10-15 minutes. The test is intended for children aged 7-18 (33). The Polish adaptation was validated by Wrocławska-Warchala, Wujcik & Kovacs was used. Reliability for the general Cronbach’s α score is 0.840–0.870 (34);
3) The Metacognition Questionnaire (MCQ-A) by Cartwright-Hatton, Mather, Illingworth, Brocki, Harrington, & Wells - is a reliable and valid instrument for assessing the severity of dysfunctional metacognitive beliefs in adolescents (aged 12-18) that affect their daily functioning (35). The first and second subscales measure “positive and negative beliefs about worry,” Subscale 3 measures “cognitive confidence,” and Subscale 4 measures “beliefs about superstition, punishment, and responsibility”. Finally, subscale 5 measures “cognitive self-consciousness,”. The test takes 10-15 minutes. The Polish adaptation were validated by Kajka & Kulik was used. Cronbach’s alpha reliability analysis for the adapted questionnaire was 0.874 (36);
4) The Perceived Stress Scale (PSS-10) by Cohen, Kamarck & Mermelstein. Consists of 10 questions and is used to measure stress based on subjective feelings related to problems and personal events, behavior, and coping with stress (37). On average, completing the test takes up to 5 minutes. The questionnaire has age norms for youth 18 years and older. The Polish adaptation was validated by Juczyński i Ogińska-Bulik. Cronbach’s alpha reliability analysis for the adapted questionnaire was 0.860 (38);
5) The Childhood Trauma Questionnaire (CTQ) by Bernstein, Fink, Handelsman & Foote. CTQ is used to assess early childhood injuries related to the experience of abuse, emotional, bodily, sexual, emotional and physical neglect. The questionnaire has age norms for adolescents (aged 12-17). Cronbach’s alpha reliability analysis for this questionnaire was 0.900 (39). The Polish translation was done by Murzyn (40);
6) The Attention and Perceptiveness Tests (APT) version 6/9- Performing the test consists in drawing the given symbols - 6, 9 among other similar ones within three minutes. The questionnaire has age norms for youth from 14 to 18 and adults over 18 years of age. The reliability of this tool measured as temporal consistency (test-retest) is 0.790. On average, completing the test takes 3 minutes. The author of the tool is Ciechanowicz (41);
7) The Rey-Osterrieth complex figure test (ROCF) was first designed by Rey and then standardized by Osterrieth. This is a neuropsychological test extensively used to investigate visuospatial constructional functions, visual graphic memory, and some aspects of planning and executive function. The questionnaire has age norms for children and adolescents from 8.6 years to 13.6 years. Cronbach’s alpha reliability analysis for this questionnaire was 0.761-0.793 (42). In the present study, participants first recreate the pattern patterns from the cards, then have a break to complete another questionnaire (CTT), and then recreate the patterns from memory after the break time (43);
8) The Yale-Brown Obsessive Compulsive Scale (CY-BOCS) by Goodman, Price, Rasmussen, Mazure, Fleischmann, Hill, Heninger, & Charney is a scale designed to measure the severity and type of symptoms in people with obsessive-compulsive disorder (44). The respondents answer 10 questions about the occurrence of symptoms in the last week. The questionnaire has age norms for children and adolescents from 12 years to 16 years. The Polish adaptation was validated by Bryńska & Wolańczyk. Cronbach’s alpha reliability analysis for this questionnaire was 0.810 (45);
9) The Colour Connection Test (CTT-1, CTT-2) by D’Elia, Satz, Uchiyama, & White. The Neuropsychological Point Matching Test measures attention, executive function, and psychomotor skills (28). It consists of connecting points with straight lines according to specific rules. The test takes 4 minutes. The Polish adaptation were validated by Łojek & Stańczak. The questionnaire has age norms for children and adolescents from 6 years to 80 years. Cronbach’s alpha reliability analysis for this questionnaire was 0.600-0.900 (46, 47);
10) The Benton Visual Retention Test (BVRT) by Benton is used to test memory, visual perception and graphomotor skills. The examined person draws the presented patterns (10 pieces) from memory or redraws them, depending on the version and methods of the research carried out by the researcher. The questionnaire has age norms for children and adolescents from 8 years to 69 years (48). Testing one version takes about 5 minutes. The Polish adaptation was validated by Jaworowska, Bac & Stańczak. The analysis of reliability as the consistency of judges’ ratings was assessed as very high (0.900-0.970) (49);
11) The Appearance Self-Rating Sheet (ASRS) - Sheet is a tool to measure the Assessment of 25 dimensions related to your body - (different parts of your body). The patient receives a visualization of the body figure on the drawing and evaluates its individual elements. The test allows you to assess the importance of appearance for patients, the assessment of satisfaction with one’s appearance, and the satisfaction index. The questionnaire has age norms for people aged 17-47. The authors of the test are Janowski, Staniewski & Jedynak. Cronbach’s alpha reliability analysis for this questionnaire was 0.719 (50).
12) The Ruminance-Reflectivity Questionnaire (RRQ) by Trapnell & Campbell *i*s a tool used to measure the intensity of processes of rumination (and reflectivity) (51). The Polish adaptation of the tool consists of 12 questions, to which the respondent responds on a 5-point scale, where 1 means - I do not agree completely, and 5 - I agree completely. The questionnaire has age norms for people aged 20-50. Cronbach’s alpha reliability analysis for this questionnaire was 0.900-0.910. The author of the test is Radoń (52);
13) The Verbal Fluency Test (VFT) by Thurstone allows for analyzing the number of spoken words beginning with the letter chosen. In the Polish version, the respondent has to enumerate as many words for the K letter as possible in 60 seconds. This is a clinical experimental test used in the diagnosis of both children and adults. Polish standards are for children aged 10-12. Reliability as temporal consistency of this tool is high, and it corresponds to a correlation coefficient of over 0.700. The Polish adaptation were validated by Borkowska, Sajewicz-Radke, Lipowska & Kalka (53);
14) Matching Familiar Figures Test (MFF) by Kagan, Rosman, Day, Albert & Phillips is used to measure conceptual tempo, that is, the relative speed with which an individual makes decisions on complex tasks (see reflection – impulsivity). It consists of the fact that the examined person has a drawing presented, and then from among 6 pictures, he has to choose the one he saw previously. The researcher counts the time in which the decision was made (54). The Polish adaptation was validated by Matczak & Wujcik. The questionnaire has age norms for people aged 9-17. Reliability analysis for reaction time showed a high index (0.85 - 0.90) and a low index for the number of errors (0.400 - 0.580) (55).

III. For assessing dietary behaviors and nutrition outcomes:

Dietary diagnosis includes 4 tests covering issues related to the severity of symptoms of eating disorders, habits and nutritional knowledge. The patient completes the questionnaires by hand, and the examination takes up to 30 minutes.

1) Eating Attitudes Test (EAT-26)- Standardized test for the detection of symptoms of eating disorders (56). It is used for screening the population at risk of anorexia, bulimia and obesity. In addition to the global score, it is also possible to analyze three EAT-26 domains, such as: (1) dietary behavior, (2) bulimia & food preoccupation, (3) oral control. The measure can be used with adolescents (13+) and adults. The EAT-26 has demonstrated good to excellent internal consistency, ranging from 0.86–0.90. Polish adaptation: Rogoza, Brytek-Matera & Garner (57);
2) Questionnaire Eating Behaviors (QEB)- The qualitative questionnaire is designed to study eating habits and opinions about food and nutrition. The first part of the questionnaire covers the frequency of consumption of selected products, the frequency of eating meals and their typical composition. In the second part of the questionnaire, there are 26 statements about food and nutrition, which enable the assessment of the level of nutritional knowledge of the respondents. The questionnaire does not include age brackets. The Polish adaptation: Wądołowska and Krusińska (58);
3) Food Intake Variety Questionnaire (FIVq)- The Variety Food Consumption Questionnaire is a qualitative food consumption frequency questionnaire. The questionnaire does not include age brackets. It is a simple tool to collect information on the consumption of (yes/no) 63 assortment groups of products. The authors of the test are Niedźwiedzka, Wądołowska (59);
4) Eating Disorder examination questionnaire (EDE-Q 6.0) by Fairburn & Beglin. The questionnaire collects data on eating behavior for the past 28 days using subscales such as: restriction, eating-related anxiety, overeating. The questionnaire has been designed for adolescents aged 14 and older (60).

IV. Scale of adverse event monitoring:

To monitor the safety and tolerability of planned neurostimulation, the tDCS Side Effects Questionnaire by Brunoni et al. will be utilized (61). Although, the application of 2mA has been considered safe in the pediatric population (62) we intend to prioritize safety considerations and meticulously monitor for potential side effects. The questionnaire is designed to standardize and improve the way adverse events are reported in clinical trials using tDCS, aiming to enhance the understanding of the safety and tolerability of this method. The self-evaluation concerns the following twelve possible tDCS side effects (headache, neck pain, scalp pain, tingling, itching, burning sensation, skin redness, sleepiness, trouble concentrating, acute mood change, others), rating on 4-point Likert scales to which extent they had experienced them (from 1: “absent” to 4: “severe”), and to which extent they attributed each experienced side effect to tDCS (from 1: “not at all” to 4: “completely”). Participants will complete the questionnaire after each tDCS session and the follow-up.

V. For biological outcomes:

A venous blood sample (20 ml) after overnight fasting will be obtained from all participants. The standard laboratory tests (complete blood count: CBC, as a marker of nutritional status) will be run by the hospital laboratory. We will examine additional parameters that are influenced by nutritional status:

1. ferritin;
2. thyroid stimulating hormone (TSH), free triiodothyronine (fT3), free thyroxine (fT4);
3. phosphorus, magnesium and calcium;
4. markers of food intake regulation (plasma leptin and serum visfatin).
5. neurotrophins (serum brain-derived neurotrophic factor: BDNF, nerve growth factor: NGF, neurotrophin 3 and 4).
6. marker of psychological stress: serum cortisol.

The part of the blood drawn will be centrifuged to obtain serum and plasma, pipette and frozen at -80°C for further analysis. All analyses will be performed via enzyme-linked immunosorbent assay (ELISA).

VI. For anthropometric data:

Body weight will be measured, and body composition will be analyzed using the electrical bioimpedance method (BIA). Measurements will be conducted three times at each stage of the study using the Impedimed device. During the examination, patients will be in a supine position with disposable electrodes placed on the left wrist and ankle of the left leg. The device scans 256 frequencies between 3 kHz and 1000 kHz and using Cole’s modeling with Hanai mixture theory, total body water, extracellular fluid, and intracellular fluid are determined. From there, fat-free mass and fat mass are calculated on the device.

VII. Neurophysiological data:

The protocol implies two EEG assessments: before the first tDSC (V1) session and after the last tDCS session (V2). The EEG measurement will take place in a well-lit, quiet room prepared for EEG examinations. A 21 Ag/AgCl electrodes cap is placed on the subject’s head, and the electrodes are covered with a gel to improve electrical conductivity. Additionally, ear clip cup electrodes will be used. The cap is additionally tightened with an elastic belt. The recording is preceded by verifying the impedance which will be kept below 5 kΩ for all electrodes. A resting-state recording with eyes closed will be performed for 10-15 minutes. Both EEG evaluations will be conducted with the same equipment and procedure. The recording will include 10-20 system of electrodes displacement with a sampling frequency of 256 Hz. After recording, the data will be band-pass filtered from 4 to 45 Hz with an active notch filter set at 50 Hz. The data will be exported in ASCII format. In further steps, EEG re-coded files will be imported to EEGLAB (63) open-source toolbox for MATLAB (Mathworks, Inc). Recordings will be cleared from artifacts in two ways, first by visual inspection by certified clinical neurophysiologist, and second using ASR and ICA algorithms. A pre-set relative amplitude values will be set as theta (4-8Hz), low-alpha (8-10.5Hz), high alpha (10.5-13Hz), beta (13-30Hz), and gamma (30-45Hz). Time-frequency analysis will bring outcomes regarding dominant frequency band power and the measurement of each frequency amplitude. This analysis will be conducted using globally averaged recordings, and will consider both the left and right hemispheres, as well as anterior asymmetry. Next, to assess signal-based functional connectivity, a weighted phase lag index (wPLI) will be used due to its consistency at a specific time and frequency examined across a pair of electrodes that minimizes the impact of volume conduction (64). FC will be analyzed in each frequency individually, with regard to its global strength and variability.

### Outcomes measures

#### Primary outcome measure

We hypothesize that the application of active tDCS will result in changes in the strength and variability of functional connectivity, with particular alterations in alpha (increase) and beta (decrease) frequencies, which will clinically lead to a reduction in the severity of psychopathological symptoms associated with AN, measured by the change in the EAT-26 scores between visit V1 (baseline) and V2 (end of stimulation after 3 weeks) as a primary outcome.

#### Secondary outcome measures

For the purpose of evaluating the effectiveness of tDCS in treating AN, a range of secondary outcome measures will also be utilized, defined as changes in: 1. anthropometric measurements (in this BMI), 2. brain bioelectric activity, 3. nutritional status, 4. mood, 5. neurocognitive functions (attention, memory, and some aspects of planning, verbal fluency, and another executive function), 6. psychological measurements connected with: dysfunctional metacognitive beliefs, other aspects related to thinking such as reflectivity, impulsivity, rumination, obsessive-compulsive symptoms, stress levels and assessment of early childhood injuries related to the experience of abuse, emotional, bodily, sexual, emotional, and physical neglect, and self-assessment of the appearance of one’s body, 7. selected biological markers related to stress, food intake, inflammation and neurotrophins.

As regards the neurophysiological measures, we expect that a series of the tDCS sessions will be associated with changes in three aspects: 1. reduction of lower frequencies (delta and theta) in the dominant frequency spectrogram accompanied with an increase in the power of alpha and beta frequencies, 2. modification of alpha anterior asymmetry patterns associated with affective functioning towards less right-hemisphere-oriented and more left-hemisphere oriented pattern, 3. changes in the strength and variability of functional connectivity, with particular alterations regarding alpha (increase) and beta (decrease) frequencies, what is associated with the ability to gain more relaxed and less tensive states recorded during resting-state condition. The neurophysiological examination will be conducted during the V1 and V2 meetings.

Secondary psychological outcome measures will be gathered during the initial assessment (V1) and V2, while nutritional data will be collected at V1, V2, and V3 after follow-up. Biological outcomes will be recorded at V1 and V2, with additional measurements of BMI and BIA analysis also at V3. Neurophysiological outcome measures will be obtained during V1 and V2.

### Study timeline

This study will consist of four visits:

V0: Screening visit to screen and enroll eligible patients into the study (1 to 2 days before the baseline visit).
V1: Baseline visit 1 day (before intervention) to randomize patients, conduct assessments using questionnaires, perform a body composition analysis, take blood samples and perform an EEG test;
V2: Visit 2, up to 3 days after the end of the intervention period, after three weeks ( ± 1 days) from baseline;
V3: End of Study Visit to complete all procedures in this study, after 5 weeks ( ± 7 days) from baseline visit.

The total duration from the screening to the end of the study will be 6 weeks at maximum.

Psychological assessments: 1. Beck Depression Inventory (BDI), 2. The Children’s Depression Inventory 2 (CDI 2), 3. The Meta-Cognitions Questionnaire (MCQ-A), 4. Perceived Stress Scale (PSS-10), 5. Childhood Trauma Questionnaire (CTQ), 6. The Attention and Perceptiveness Tests version 6/9, 7. The Rey-Osterrieth complex figure test (ROCF), 8. The Yale-Brown Obsessive Compulsive Scale (CY-BOCS), 9. Color connection test (CTT-1, CTT-2), 10. The Benton Visual Retention Test (BVRT),11. The Appearance Self-Rating Sheet (ASRS),12. The Ruminance-Reflectivity Questionnaire (RRQ), 13. The Verbal Fluency Test (VFT), 14. Matching Familiar Figures Test (MFF).

Neuropsychological assessment: EEG.

Dietary behaviors and nutrition assessments: 1. Eating Attitudes Test (EAT-26), 2. Questionnaire Eating Behaviors (QEB), 3. Food Intake Variety Questionnaire (FIVq), 4. Eating Disorder Examination Questionnaire (EDE-Q 6.0).

Biological assessments: 1. Complete blood count (CBC), 2. Ferritin, 3. TSH, 4. FT3, 5. FT4, 6. Phosphorus, 7.Magnesium, 8. Calcium, 9. Cortisol, 10. Neurotrophins, 11. Leptin, 12. Visfatin.

### Sample size calculation

The number of studies that examined the effect of tDCS in patients with AN group is relatively small. To calculate the sample size, we analyzed the number of patients in the general population (patients suffering from AN in Poland) (65) and previous trials conducted by other authors (18), which reported improvement in EAT-26 scores. The statistical power was estimated at 80% to detect a difference in means of EAT-26. The baseline EAT-26 points was estimated at 72.7, with a 20-point improvement (18). To account for a dropout rate of 20%, a total of 50 individuals will be included to achieve a target number of 40 participants. Patients will be recruited for the study and randomized into two subgroups. The recruitment target was determined to ensure statistical power, which allows confirmation of the differences in the primary outcomes with an approximately 95% probability of correctly rejecting the null hypothesis. The calculation of sample size was performed using the STATISTICA software (StatSoft) and online sample size calculator (66).

### Recruitments

The participants of the study will be recruited from consecutively admitted patients for hospital treatment at the University I Department of Psychiatry, Psychotherapy and Early Intervention, Child and Adolescent Psychiatry Unit in Lublin, Poland.

According to the Eating Disorders Therapy Program adopted by our Department, patients with AN have an adaptation period of up to three days. On the fourth day, patients will be informed in detail on the course of the study, all study procedures, potential risks, and the benefits of each intervention. A written document outlining the study will be provided to all participants. This document will include the objectives and rationale of the research, assurances of voluntary participation, the study schedule, potential hazards, the procedures for maintaining the confidentiality of personal data, and the participants’ right to discontinue participation in the study at any moment without the necessity to provide a justification. The next step involves obtaining written informed consent for participation in the study from the participant or participant and parent/legal guardian.

### Allocation

The patients included in the study will be randomly assigned to one of two subgroups, independently of examined variables, during the entry of the study: Active group - AG (tDCS group, n=25) and Sham group - SG (placebo group, n=25).

Eligible patients will be randomized in equal proportions between the AG and SG arm. A random sequence generator (Urbaniak GC, Plous S. Research Randomizer (Version 4.0) 2013) will be used to randomize active versus sham arms. The allocation to study groups will be performed by a researcher not involved in the stimulation and assessment of patients.

### Blinding

The researchers, participants, and their parents will be unaware of the group allocation as part of the blinding process. Moreover, data for each measure will be obtained by blinded experimenters and subsequently entered into secure data files to maintain confidentiality and protect the integrity of the study. The randomization will be performed using a random sequence generator and the results of allocation will be saved in a spreadsheet. The research assistant will generate randomization identification numbers for each participant based on the obtained data. The stimulation condition (duration, electric current, active or sham therapy) must be entered into the device to generate unique codes. The obtained number, together with the patient’s ID, will be written by the technical worker in an online spreadsheet. The study research coordinator will download the code from the prepared online sheet each time before the tDCS session and mark the used code.

## Data collection methods

To evaluate the efficacy of the intervention on the symptoms of patients with AN, the following changes will be determined:

1. Using paper-pencil questionnaires:

a) the severity of depression symptoms between V1 and V2 measurement (BDI total score/CDI2 total score);

b) the severity of symptoms of eating disorders and inappropriate eating behaviors (EAT-26 total score, QEB total score, EDE-Q 6.0 total score, FIVq total score);

c) neurocognitive functions between V1 and V2 measurement (total score: APT, ROCF, CTT-1, CTT-2, BVMT, VFT).

d) psychological measurements connected with dysfunctional metacognitive beliefs, other aspects related to thinking (such as reflectivity, impulsivity, rumination) and obsessive-compulsive symptoms, stress levels and assessment of early childhood injuries related to the experience of abuse, emotional, bodily, sexual, emotional, and physical neglect, and self-assessment of the appearance of one’s body between V1 and V2 measurement (total score: MCQ-A, RRQ, MTT, CY-BOCS, PSS-10; CTQ, ASRS),.

2. To evaluate the effect of the intervention on physiological parameters, the following changes will be measured:

a) In serum, from the baseline (V1) to the end of the stimulation (V2), levels of:

CBC, ferritin, TSH, FT3, FT4, electrolytes: phosphorus, magnesium and calcium, HPA axis biomarker: cortisol, markers of food intake regulation (plasma leptin and serum visfatin), neurotrophins (serum brain-derived neurotrophic factor: BDNF, nerve growth factor: NGF, neurotrophin 3 and 4.

Blood will be drawn by a qualified hospital staff member, and then a portion of it will be analyzed in the hospital laboratory, while another part will be frozen for conducting ELISA analyses at the level of neurotrophins, cortisol, and food intake regulators after the completion of the group collection.

b) Anthropometric data, from the baseline (V1) to the end of the study (V3):

Body weight will be measured, and body composition will be analyzed using the electrical bioimpedance method (BIA), Impedimed SFB7 BIS Research Device will be used. Anthropometric measurements will be carried out by a dietitian.

c) Brain activity, at the baseline (V1) and at the end of the stimulation (V2): EEG. The examination and analysis will be conducted by a qualified employee of the clinical neuropsychology laboratory.

### Statistical methods

The statistical analysis will be performed by the Statistica package, version 13.0 (StatSoft, TIBCO Software Inc.). Before analysis, the Shapiro-Wilk test will be applied to examine the distribution of examined variables where normality will be assumed if p > 0.05. The chi-square test will be used to calculate differences in the qualitative data at baseline between groups. Depending on data distributions, the between-group comparisons will be performed using the ANOVA or Kruskal-Wallis rank-based nonparametric test for quantitative outcomes. To compare groups between baseline and endpoint assessments and follow-up assessments, repeated measures of ANOVA or aligned rank-transform ANOVA and McNemar’s test will be conducted. All analyses will be performed according to the ICH E9 Statistical Principles for Clinical Trials (67). Bonferroni correction of obtained significance levels will be used to correct for multiple comparisons. The additional characteristics of the participants and dropouts will be performed for an additional assessment of possible non-random differences.

### Data monitoring and collection

Z.R will be responsible for collecting nutrition-related data and anthropometric measurements, P.S will conduct psychological tests, P.K will be responsible for collecting and processing neurophysiological data. The study will be overseen by three supervisors (H.K-J, J.R, N.K): two supervisors (H.K-J, J.R) with collective expertise in psychiatry, nutrition research, eating disorders and statistics, one supervisor (N.K) will be responsible for psychological assessment.

## Discussion

AN is a serious and incapacitating eating disorder characterized by a high incidence and mortality rate. Existing treatment methods have demonstrated moderate effectiveness. Neuroimaging studies provide valuable insights for developing contemporary therapeutic approaches, such as tDCS, a non-invasive technique for neuromodulation in the brain.

Research conducted so far suggested benefits for core eating disorder symptoms of tDCS in AN. In a double-blind RCT focusing on tDCS therapeutic potential, 43 participants with AN were randomly assigned to receive active or sham tDCS over the left DLPFC during ten 30-minute sessions. While there were no significant improvements in AN psychopathology or weight recovery compared to sham, active tDCS did decrease the need for excessive control over food intake and improved body image (17). Furthermore, three smaller open-label studies (14, 16, 18) investigated the efficacy of left DLPFC anodal stimulation in AN. However, each of the studies presents a different methodology of the applied stimulation. Costanzo et al. (16) aimed to investigate tDCS’s potential in modifying inter-hemispheric balance in adolescents with AN. The study was finished by 11 patients undergoing treatment as usual (AU), with interventions comprising 18 sessions of tDCS combined with AU or family-based therapy combined with AU. No sham was used in the study. Various measures, such as EDI, EAT-26, Body Uneasiness Test (BUT), anxiety, depressive symptoms, and BMI, were employed. Medications, including aripiprazole and SSRIs, were administered, and adverse effects like itching, burning sensations, local redness, headaches, and tingling were reported. Baumann et al. (17) conducted a double-blind, randomized controlled trial with 43 inpatients to examine the effects of 10 tDCS sessions on eating behavior, body weight, and depression in AN. Participants received either active tDCS or sham tDCS. Strumila et al. (14) conducted a pilot study with nine female AN patients, assessing the effects of tDCS during 20 sessions, with two sessions per day for two weeks. Various psychiatric medications were prescribed, and measures included EDI, EDE-Q, Body Shape Questionnaire (BSQ-34), and BDI. In a study by Khedr et al. (18), anodal tDCS (2mA) for 25 minutes over the left dorsolateral prefrontal cortex daily for ten days was conducted on a group of patients. Adverse effects of tDCS were minimal, with only transient local itching reported by two patients and no other observed adverse effects. Collectively, these studies provide insights into tDCS’s potential in treating anorexia, with varying methodologies and outcomes, while indicating a generally tolerable safety profile in the context of AN treatment.

While tDCS holds promise for treating individuals with AN, much of its potential remains untapped. Response variations among patients still pose a significant challenge. Exploring and optimizing protocols, patient selection criteria and intervention parameters is necessary. Moreover, in the author’s opinion, deepening our understanding of neurocognitive correlations and biological markers is essential. Therefore, we decided to expand our research to include biological and neurophysiological components, incorporating not only BMI but also EEG measurements and blood parameters.

It has been shown that BDNF is lower in AN patients compared to healthy controls. The neurotrophins affect the morphology and physiology of neurons and are essential regulators of eating behavior. The tDCS modulates BDNF at the molecular level (both gene and protein expression levels). The comparison of the neurotrophin concentrations with the severity of AN symptoms at baseline and the end of the study will be interesting to improve the quality and significance of our study (68, 69).

Women with AN have higher cortisol levels compared to individuals without AN. Metaanalysis finds that non-invasive brain stimulation can regulate HPA axis reactivity and cortisol release in acute stress situations. However, according to a 3-week, randomized, triple-blind pilot trial and studies with shorter duration, the tDCS stimulation does not change the cortisol levels. Contrary, some studies find that the tDCS decreases levels of psychological stress. We hypothesized that enhanced intensification of interventions through tDCS stimulation (twice daily for 25 minutes over a period of 3 weeks) can decrease cortisol serum levels (70–72).

### Strengths and weaknesses

The protocol was developed by a multidisciplinary research team of a psychiatrist, psychologist, psychotherapist and nutritionists. Thanks to this, it is possible to monitor changes occurring during the intervention across multiple dimensions. Our study emphasizes two notable strengths: the high intensity of stimulation, the inclusion of biological measurements, and a broad array of psychological tests. To the best of our knowledge, no prior studies have evaluated the impact of tDCS on neurotrophins in AN. However, it is essential to acknowledge certain limitations within our project, such as a relatively small sample size and a restricted follow-up period. Another limitation of our study is the decision to include only female participants. This choice was driven by the disparities in the prevalence of AN between the two genders (73), and assembling an equal-sized group of both men and women for the study would require a significant amount of time. Despite these weaknesses, our findings will contribute valuable insights into the potential effects of tDCS in AN and underscore the need for further research in this domain.

## Ethics statement

The study received a positive opinion of the Bioethics Committee at the Medical University of Lublin. Written informed consent to participate in this study is provided by the participants and/or participants legal guardian.

## Author contributions

ZR: Conceptualization, Investigation, Writing – original draft. JR: Methodology, Writing – review & editing. NK: Investigation, Writing – review & editing. PS: Investigation, Writing – orginal draft. PK: Methodology, Writing – review & editing. HK: Supervision, Writing – review & editing.
